# Improving participation rates by providing choice of participation mode: two randomized controlled trials

**DOI:** 10.1186/s12874-015-0021-2

**Published:** 2015-04-02

**Authors:** Naomi Heijmans, Jan van Lieshout, Michel Wensing

**Affiliations:** Radboud University Medical Centre, Nijmegen, Scientific Institute for Quality of Healthcare, PO 9101, 6500 HB Nijmegen, The Netherlands

**Keywords:** Participation rates, Patient preferences, Participation mode, Randomized controlled trial

## Abstract

**Background:**

Low participation rates reduce effective sample size, statistical power and can increase risk for selection bias. Previous research suggests that offering choice of participation mode can improve participation rates. However, few head-to-head trials compared choice of participation mode using telephone interviews and postal questionnaires as modes of interest. Aiming to explore effects of choice of participation, two randomized controlled trials were performed comparing participation rates of patients provided with and without choice of participation mode, using interviews and questionnaires as participation modes.

**Methods:**

Two trials were embedded in a larger study on cardiovascular risk management in primary care. Patients with a chronic cardiovascular condition recruited for the larger study were invited to participate in an additional survey on social networks, using invitations with and without choice of participation mode. Primary outcome was participation rate. Other outcomes of interest were participation rate conditional on willingness to participate, and initial willingness to participate. In trial 1 we compared outcomes after choice of participation mode (interview or questionnaire) with invitations for participation in a telephone interview. In Trial 2 results for choice of participation mode were compared with postal questionnaires.

**Results:**

In Trial 1 no differences were found in participation rates (65% vs 66%, p = 0.853) although conditional participation rate was highest for interviews (90% vs 72%, p < .01). Initial willingness to participate was higher when choice of participation mode was provided (90% versus 73%, p < .01). In Trial 2 participation rate and conditional participation rate was higher when choice of participation mode was provided (59% vs 46%, p < .01 and 66% vs 53%, p < .01, respectively). No differences were found for initial willingness to participate (90% vs 86%, p = 0.146).

**Conclusion:**

Offering choice of participation mode had benefit on participation rates compared to invitations to participate in questionnaires, but not when compared to invitations to participate in telephone interviews.

**Trial registration:**

Current Controlled Trials ISRCTN89237105.

## Background

Low participation rates reduce effective sample size, statistical power and can increase risk for selection bias. Appealing evidence suggests that offering potential participants choice of participation mode may improve response rates. However, few head-to-head trials compared telephone interviews and postal questionnaires as participation modes of interest.

In recent years, mixed mode designs for data collection became increasingly popular. The idea is that participants who are lost when offering a particular participation mode, can still be included by providing an alternative mode. Previous research reported that respondents do have mode preferences [1-3], but evidence on whether mode preference actually predicts participation remains inconclusive [4]. Only a few studies investigated participation rates, comparing participants provided with and without choice of mode, and using telephone interviews and postal questionnaires as participation modes of interest. A study on census questionnaires compared response rates among several panels of households, provided with and without choice to respond by telephone or by mail. This study did not identify enhanced response rates when comparing the households panels provided with choice compared to households who were only allowed to respond by mail [5]. Another study in cancer survivors reported completion rates calculated for patients who provided consent to participate in the study and found improved completion rates for patients allowed to choose a participation mode compared to those not provided with choice and participating in telephonic interviews or postal questionnaires as designated by the researchers. However, differences did not reach statistical significance [6]. So, evidence for choice of participation mode using telephone interviews and postal questionnaires as choice options is scarce and mixed.

The vast part of previous research on choice of participation mode compared a traditional mode (e.g. face-to-face interview, postal questionnaires) with web-based modes (e.g. email, online questionnaires) as choice options and found that response rates of those allowed to choose a participation mode declined [7-9]. Such results may be explained by a cognitive burden of choosing, technical problems, and deciding to participate but failing to do so [7]. The latter may occur as responding on web-based options involves a break in response processes, e.g. a switch in behavior is required when moving from sorting and responding emails to filling out questionnaires [7].

Although web-based participation modes may have their attractiveness (e.g. reduced costs, less missing data) it may not be suitable for all groups. For instance, elderly individuals with chronic diseases may be less likely to participate [10,11]. In the current research, we aim to investigate the effect of providing choice of participation mode in a survey of social information exchange networks in patients with chronic cardiovascular conditions. This group is typically an older one [12] with a lower use of the Internet. Data from 2013 showed that only 55% of persons between 65–75 years in the Netherlands used Internet on a daily basis, which is substantially lower compared to 87% of persons between 12–65 years. For persons 75 years and older, this percentages drops to 20% [13]. Therefore, participation modes of interest were telephone interviews and postal questionnaires.

In this study, in the following to be referred to as SNS (Social Network Study), two randomized controlled trials (RCT) were performed. Patients with a chronic cardiovascular condition were randomly allocated to a choice and no-choice arm for participation mode. Considering the scarce and mixed literature, we aim to explore effects of providing choice of participation mode. Assuming that providing choice of mode will retain patients who are lost when a single provided mode is provided, we will test the following hypothesis:

H1: Participation rates will be higher when potential participants are provided with choice of participation mode, compared to those of patients provided with only one participation mode.

Two trials were performed, varying participation mode in the no-choice arm. This approach was chosen to exclude the possibility that results would be biased by the possibility that the no-choice arm would simply represent a less popular mode. In trial 1 we compared participation rates of choice of participation mode with invitations for a telephone interview. In trial 2 the choice arm was compared with an invitation for a postal questionnaire. Additionally, using data from patients from choice arms and expecting that patients would voice preferences for a particular participation mode, mode preference will be determined.

## Methods

### Design and study population

**Figure 1:**
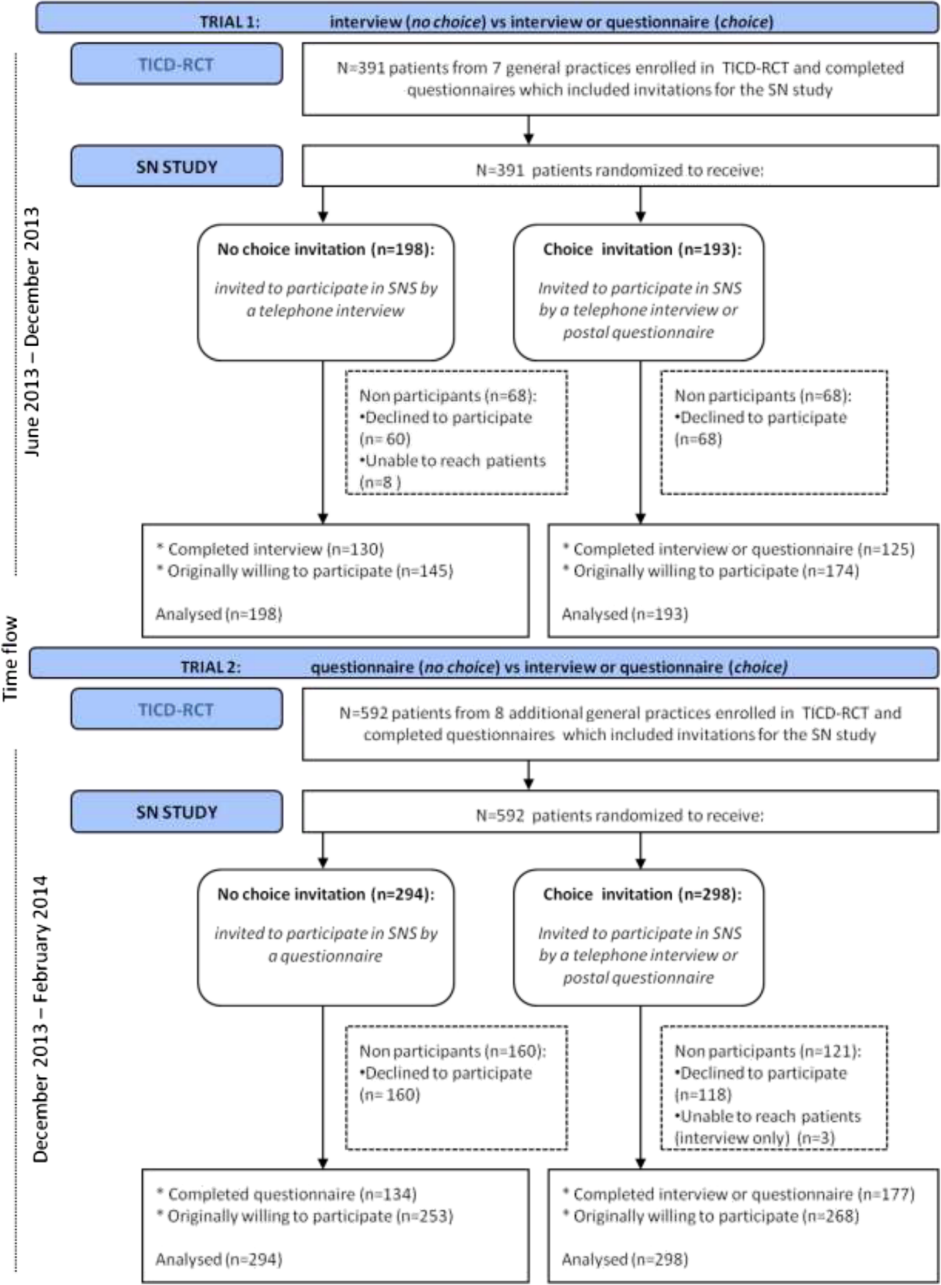
**Study flow.**

The SNS (ISRCTN89237105) is part of the ‘Tailored Implementation for Chronic Diseases’ (TICD)-project [14] and was an observational study on social networks of information sharing from patients involved in cardiovascular risk management (CVRM) [15]. Within the SNS, two RCTs on choice of participation mode were embedded. The SNS and its RCTs were, in turn, performed parallel to a larger two-arm RCT (NTR4069), also part of the TICD project (see Figure 1). In this paper we will refer to the larger RCT as the ‘TICD-RCT’. The TICD-RCT aimed at improving CVRM in primary care by enhancing professional performance of practice nurses and included a random sample of general practices from several geographical areas in the Netherlands. For specific details on the TICD-RCT we refer to its study protocol which has been published elsewhere [16].

Potential participants for the SNS were identified from the TICD-RCT. Both patients with high risk for cardiovascular disease (CVD) and established CVD were included. International Classification of Primary Care (ICPC) codes were used to extract eligible patients from medical records from general practices. Extraction was performed by research assistants in cooperation with practice nurses. Eligible patients were 18 years or older and capable of providing informed consent, exclusion criteria consisted of: diabetes mellitus, pregnancy and lactation, terminal illness, cognitive impairments, and poor language skills. To exclude patients with diabetes ICPC codes were used, other exclusion criteria were assessed by practice nurses.

### Data collection procedures

Participants for the SNS were approached using differently formatted invitations enclosed at the end of postal questionnaire booklets sent on behalf of the TICD-RCT at baseline of the TICD-RCT intervention program (see also Figure 1). TICD-RCT questionnaires mainly contained questions on health-related lifestyle. Invitations for the SNS contained a concise explanation on the study purpose. An informed consent form was enclosed, explaining that patients consented to be approached for a baseline and follow up measure after six months. No incentives for participation were offered. On invitations with choice, patients could indicate their preferred participation mode by ticking one of two boxes for ‘Yes, I agree to participate in a telephone interview’ and ‘Yes, I agree to participate by a postal questionnaire’.

### Randomization

Randomization to the choice or no-choice arm of the SNS was performed per general practice, using a computer assisted procedure and was performed by an independent research assistant. Patients were not informed about study arms of both the SNS and the TICD-RCT.

Two trials were performed subsequently, following inclusion procedures of the TICD-RCT. Subsequent rather than simultaneous conduct of the two trials matched best with the logistics of running the TICD-RCT.

### Trial 1: telephone interview versus choice of participation mode

Invitations for trial 1 were sent from June 2013 till November 2013. During this period, patients were randomly invited to participate in a telephone interview on their social networks (the no-choice arm) or invited to participate in either a telephone interview or postal questionnaire (the choice-arm). In trial 1 patients from seven general practices were invited for the TICD-RCT. Of these, three general practices were randomized to the control arm of the TICD-RCT and four to its intervention arm. A total of 391 patients (mean patients per practice: 56, SD 12.9) completed questionnaire booklets for the program evaluation and thus received invitations for the SNS in trial 1.

### Trial 2: postal questionnaire versus choice of participation mode

Invitations for trial 2 were sent from December 2013 till February 2014. Patients were randomized to participate in the SNS in a postal questionnaire (the no-choice arm) or provided with choice for a telephone interview or a postal questionnaire (the choice-arm). During this trial, inclusion procedures to the TICD-RCT needed to be adjusted because too few patients were included to achieve the TICD-RCTs’ aimed sample [16]. Therefore, the number of patients receiving questionnaires was increased by 25%. A total of 592 patients (mean patients per practice: 74, SD 5.9) from eight additional general practices (three in the control and five in the intervention arm of the TICD-RCT) received invitations for trial 2.

In both trials, telephone interviews were held and postal questionnaires were sent up to a maximum of two months after receipt of completed informed consent forms. This interval of two months was needed to include patients who were difficult to reach for interviews and due to logistical constraints in the TICD-RCT. For telephone interviews, a maximum of ten attempts were made before considering patients as unable to reach. Patients were contacted for telephone interviews during office hours and in early evening (up to 20.00 pm). For postal questionnaires, patients were provided with a postage-paid envelope to return their completed questionnaires.

For patients in choice arms of the two trials, all who indicated a preferred participation mode received an interview or questionnaire according to the stated preference. For patients who indicated to be willing to participate by both modes (i.e. ticked both the boxes for telephonic interview and postal questionnaire), all were sent a postal questionnaire. This approach was chosen for reasons of feasibility.

### The telephone interview & postal questionnaire

Telephone interviews and postal questionnaires contained identical questions, regardless of study arm for the SNS and TICD-RCT. Total number of main questions was five, including sub questions the total number of questions was 45. The SNS included questions on 1) information sharing with health care providers and persons from patients’ personal networks, and 2) on persons that patients considered to be important for handling their condition or disease. Questions were tailored to general practice. In this way, for items on information sharing with health care providers, names and disciplines of persons from patients’ general practices were prelisted with space for additional names and disciplines if needed.

Note that the number of questions to be completed was dependent on the composition of patients’ networks, so that patients with smaller networks needed to complete fewer questions. Patients with missing data on questions that they could have completed given their answers on other questions, were considered as partial completions. Patients were considered as withdrawn when they refused to participate when being contacted for the interview or when they contacted the research team (either by telephone, email, or letters) about their non participation or when they failed to return their questionnaire within two months.

Mean duration of interviews was 15.7 minutes (SD 6.91). Number of pages for the questionnaire was twelve.

### Ethical approval

The Medical Ethical Committee of Radboud University Nijmegen Medical Centre has waived approval for the social network study and its associated response trials [15], as well as the TICD-RCT [16].

### Measures & outcomes

The primary outcome was participation rate. Secondary outcomes consisted of conditional participation rate, willingness to participate, and mode preference. Definitions for all outcomes are summarized in Table 1.

**Table 1:** **Definitions of outcomes**

**Outcome**	**Definition**
Participation rate	Completed and partially completed SNS interview or questionnaire
	Number of participants in TICD-RCT
Conditional participation rate	Completed and partially completed SNS interview or questionnaire
	Willing to participate in SNS
Willing to participate	Willing to participate in SNS
	Number of participants in TICD-RCT
Mode preference	Willing to participate in SNS by a particular mode
	Received a choice-format invitation and willing to participate in SNS

### Participation rate

Was defined as the percentage of *patients who actually participated* in the SNS. That is the total number of patients who had completed and partially completed an interview or questionnaire for the SNS divided by the total number of participants in the TICD-RCT (conform to AAPOR RR2 [17]).

### Conditional participation rate

The invitation procedures of this study allowed for determining what percentage of patients participated in the SNS, *given that they were willing to participate*. This secondary outcome is defined as the total number of patients who had completed and partially completed an interview or questionnaire for the SNS divided by the total number of patients willing to participate in the SNS (conform to AAPOR COOP2 [17]).

### Willingness to participate

Was defined as the percentage of patients *initially willing to participate* in the SNS. That is the total number of patients willing to participate in the SNS divided by the number of participants in the TICD-RCT. This definition is conform to AAPOR RR2 [17] with the number of patients accepting invitations to participate in the SNS in the numerator.

### Mode preference

Additionally, we determined preference for participation mode for patients in the choice arms of the SNS. Mode preference was determined as the total number of patients willing to participate by a specific mode (telephone interview, postal questionnaire, or both) divided by the number of participants who were provided choice of participation mode and who accepted to participate in the SNS.

### Sample size & statistical analysis

Assuming a two-tailed alpha of 0.05, 80% power, a response rate of 50% in the no-choice arm and 65% in the choice arm, we estimated we needed to include a total of 338 patients in each trial [15].

Data were analyzed using SPSS version 20. All analyses were based on intention to treat. Chi square tests were used to examine differences in participation rates, conditional participation rates, and willingness to participate for the different invitation formats. Relative risks (RR) and 95% confidence intervals are reported for the effectiveness on participation rates, conditional participation rates, and willingness to participate. Reference categories consisted of ‘decided not to participate’ for participation rates and conditional rates and of ‘unwilling to participate’ for willingness to participate.

Two types of sensitivity analyses were performed. First, effect of choice of participation mode was controlled for general practice clustering, TICD-RCT trial arm, and patient characteristics (sex, age, and patient group: high risk or CVD). For this means, we estimated logistic models with participation rate, conditional participation rate, and willingness to participate in trial 1 and 2 as outcomes, using generalized estimating equations (GEE) with general practice as subject variable. The working correlation structure was specified as exchangeable, and robust sandwich estimators were used. Second, patients from choice arms who indicated to be willing to participate in the SNS by both modes, all were sent questionnaires. To examine whether this approach influenced outcomes, we again used chi square tests to examine differences in participation rate, conditional participation rates, and willingness to participates, excluding patients without preference of participation mode.

## Results

### Description of sample

Table 2 provides descriptive characteristics of the samples in each trial. Participants in trial 1 had a mean age of 72 years, 38% female, and 60% were at high risk for CVD. In trial 2 mean age was 73 years, 32% female, and 58% were at high risk for CVD.

**Table 2:** **Sample characteristics**

	**Trial 1**			**Trial 2**		
**Trial arm**	**No choice**	**Choice**	**Total**	**No choice**	**Choice**	**Total**
Participants	198	193	391	294	298	592
Female	72 (36%)	77 (40%)	149 (38%)	95 (32%)	97 (33%)	192 (32%)
Age	71.8 (SD 9.2)	73.1 (SD 9.9)	72 (SD 9.6)	72.9 (SD 8.8)	72.3 (SD 10.3)	73 (SD 9.6)
HR	118 (59.6%)	115 (60%)	233 (60%)	168 (57%)	173 (58%)	341 (58%)
CVD	80 (40.4%)	78 (40%)	158 (40%)	126 (43%)	125 (42%)	251 (42%)

Abbreviations: HR = high risk for CVD, CVD = cardiovascular disease.

### TRIAL 1: No choice (telephone interview) versus choice of participation mode

Results from Trial 1, comparing outcomes of patients invited to participate in the SNS in a telephone interview and provided with choice of participation mode (n = 391) are summarized in Table 3.

**Table 3:** **Willingness to participate, participation rates, and conditional participation rates in trial 1 and 2**

***Invitation formats***	***No choice***	***Choice***				
***TRIAL 1***	**interview (n = 198)**	**interview or questionnaire (n = 193)**	***X*** ^***2***^	***df***	***p***	**RR (95%CI)**
Participation rate	130 (66%)	125 (65%)	0.03	1	0.853	0.98 (0.74 - 1.28)
Conditional participation rate	130 (90%)	125 (72%)	15.65	1	<.01	0.37 (0.22 - 0.63)
Willing to participate	145 (73%)	174 (90%)	18.63	1	<.01	2.72 (1.67 - 4.42)
	***No choice***	***Choice***				
***TRIAL 2***	questionnaire (n = 294)	interview or questionnaire (n = 298)	*X* ^*2*^	*df*	*p*	RR (95%CI)
Participation rate	134 (46%)	177 (59%)	11.33	1	<.01	1.34 (1.13 - 1.59)
Conditional participation rate	134 (53%)	177 (66%)	9.25	1	<.01	1.39 (1.12 - 1.71)
Willing to participate	253 (86%)	268 (90%)	2.11	1	0.146	1.39 (0.89 - 2.16)

Abbreviations: X^2^ = chi square, RR = relative risk.

### Participation rates

Participation rates of patients with and without choice of participation mode did not differ (RR 0.98, 95% CI:0.74 – 1.28); 65% of patients who choose their preferred participation mode actually participated, compared to 66% of patients who were not allowed to choose participation mode (X^2^ 0.03 (1), p = .853).

### Conditional participation rates

conditional participation rates (that is the percentage of patients actually participating, provided that they were willing to participate) differed, with fewer (72%) patients willing to participate by means of a participation mode according to their preference actually doing so and more (90%) patients willing to participate in an interview actually doing so (RR 0.37, 95%CI: 0.22 – 0.63). This 18% difference in participation rates was statistically significant (X^2^ 15.654 (1), p < .01).

### Willingness to participate

In trial 1, more patients were initially willing to participate in the network study when allowed to choose a participation mode compared to patients invited for an interview (RR 2.72, 95%CI: 1.67 – 4.42) ; 90% of patients allowed to choose their preferred participation mode were willing to participate, compared to 73% patients invited to participate in an interview. This 17% difference was significant: X^2^ 18.631 (1) p < .01).

### TRIAL 2: No choice (postal questionnaire) versus choice of participation mode

Results from Trial 2, comparing outcomes of patients invited to participate in the SNS by a postal questionnaire and provided with choice of participation mode (n = 592) are summarized in Table 3.

### Participation rates

Participation rate of patients who were allowed to choose participation mode was higher than that of patients who were not allowed to choose their participation mode (RR 1.34, 95% CI: 1.13 – 1.59); 59% versus 46% respectively. This 13% difference was significant (X^2^ 11.33 (1), p < .01).

### Conditional participation rates

Conditional participation rate was higher for patients who were allowed to choose participation mode; 66% versus 53% (RR 1.39, 95% CI: 1.12 – 1.71). This 13% difference was significant (X^2^ 9.25 (1), p < .01).

### Willingness to participate

In trial 2, initial willingness to participate did not differ (RR 1.39, 95% CI: 0.89 – 2.16); 90% of patients who received a choice format invitation were willing to participate, whereas 86% of patients who were invited to participate via a questionnaire were willing to participate. This 4% difference was not statistically significant (X^2^ 2.11 (1), p = .146).

### Mode preference

For patients from the choice arms of the two trials and who accepted the invitation to participate in the SNS, mode preference was inferred (see Table 4).

**Table 4:** **Mode preference**

	***Participation mode:***	**Interview**	**Questionnaire**	No preference*	***Total***
**Trial 1**	Willing to participate	37 (21%)	57 (33%)	80 (46%)	174
	Participated	37 (100%)	35 (61%)	53 (66%)	125 (72%)
**Trial 2**	Willing to participate	32 (12%)	127 (47%)	109 (41%)	268
	Participated	27 (84%)	83 (65%)	67 (61%)	177 (66%)

*patients willing to participate by both participation modes were considered to have no preference for mode.

In trial 1, a total of 174 patients from the choice arm was willing to participate in the SNS. Of these patients, 46% were willing to participate in both modes. 54% of patients preferred one participation mode of which 21% preferred the telephone interview and 33% preferred the postal questionnaire.

In trial 2, a total of 268 patients from the choice arm was willing to participate in the SNS. Of these patients, 41% were willing to participate in both modes. 59% of patients preferred one participation mode of which 12% preferred the telephone interview and 47% preferred the postal questionnaire.

Conditional participation rates of patients from the choice arm, stratified for chosen participation mode, were highest for interviews with a 100% participation rate in trial 1 and 84% in trial 2.

### Sensitivity analyses

#### GEE analyses

Table 5 provides results of three logistic models using GEE with participation rate, conditional participation rate, and willingness to participate as outcomes while controlling the effect of choice of participation mode in trial 1 (interview vs. choice of participation mode) for general practice clustering, TICD-RCT trial arm, and several patient characteristics (sex, age, and patient group). Effects of choice of participation mode remained stable for all outcomes. In Table 6 results are presented for trial 2 (questionnaire vs. choice of participation mode). Effects of choice of participation mode remained stable for participation rate and conditional participation rate. Different from the chi square test, the effect of choice of participation mode did reach statistical significance (OR = 1.42, p < .001) in the analysis for willingness to participate.

**Table 5:** **Logistic regression models using GEE for outcomes in trial 1: no choice (interview) versus choice of participation mode**

	**Participation rate**		**Conditional participation rate**		**Willingness to participate**	
	**OR**	**(95% CI)**	**OR**	**(95% CI)**	**OR**	**(95% CI)**
SNS choice arm	0.99	(0.68-1.44)	0.27***	*(0.17-0.44)*	3.52***	*(2.75-4.51)*
SNS no-choice arm						
TICD-RCT intervention arm	0.84	(0.62-1.14)	0.60*	*(0.40-0.90)*	1.14	(0.64-2.02)
TICD-RCT control arm						
Patient group: CVD	1.16	(0.75-1.79)	1.37	(0.62-3.04)	1.25**	*(1.07-1.46)*
Patient Group: high risk						
Female	0.96	(0.73-1.26)	1.12	(0.59-2.12)	0.85	(0.64-1.13)
Male						
Age	0.99	(0.98-1.02)	1.03*	*(1.01-1.06)*	0.97	(0.94-1.002)

***p < .001 **p < .01 *p < .05, OR = odds ratio, estimated intercepts omitted from table.

**Table 6:** **Logistic regression models using GEE for outcomes in trial 2: no choice (postal questionnaire) versus choice of participation mode**

	**Participation rate**		**Conditional participation rate**		**Willingness to participate**	
	**OR**	**(95% CI)**	**OR**	**(95% CI)**	**OR**	**(95% CI)**
SNS choice arm	1.81***	*(1.37-2.39)*	1.81**	*(1.29-2.55)*	1.42***	*(1.22-1.65)*
SNS no-choice arm						
TICD-RCT intervention arm	0.95	(0.53-1.73)	0.98	(0.50-1.90)	0.80	(0.54-1.19)
TICD-RCT control arm						
Patient group: CVD	0.95	(0.70-1.30)	0.93	(0.60-1.43)	1.12	(0.65-1.94)
Patient Group: high risk						
Female	0.87	(0.68-1.10)	0.92	(0.67-1.25)	0.86	(0.51-1.44)
Male						
Age	1.01	(0.99-1.02)	1.02*	*(1.001-1.03)*	0.97*	*(0.94-0.99)*

***p < .001 **p < .01 *p < .05, OR = odds ratio, estimated intercepts omitted from table.

#### Examining choice of participation mode excluding patients without mode preference

In these analyses (see also Table 7) comparisons for participation rates, conditional participation rates, and willingness to participate were repeated excluding patients from choice arms of both trials who did not express preference for a participation mode. Results were similar to those of the main analyses as reported in Table 3.

**Table 7:** **Sensitivity analyses excluding patients without mode preference**

***Invitation formats***	***No choice***	***Choice***				
***TRIAL 1***	**Interview (n = 198)**	**Interview or questionnaire (n = 113)**	***X*** ^***2***^	***df***	***p***	**RR (95%CI)**
Participation rate	130 (66%)	72 (64%)	0.12	1	0.73	0.95 (0.69 - 1.29)
Conditional participation rate	130 (90%)	72 (77%)	7.43	1	<.01	0.44 (0.24 - 0.81)
Willing to participate	145 (73%)	94 (83%)	4.006	1	<.05	1.59 (1.00 - 2.55)
	***No choice***	***Choice***				
***TRIAL 2***	**Questionnaire (n = 294)**	**Interview or questionnaire (n = 189)**	***X*** ^***2***^	***df***	***p***	**RR (95%CI)**
Participation rate	134 (46%)	110 (58%)	7.33	1	<.01	1.30 (1.07 - 1.59)
Conditional participation rate	134 (53%)	110 (69%)	10.63	1	<.01	1.53 (1.17 - 1.99)
Willing to participate	253 (86%)	159 (84%)	0.34	1	0.56	0.88 (0.57 - 1.36)

## Discussion

In this study we examined whether participation in a survey study can be improved by providing choice of participation mode. Results were mixed. In trial 1 patients offered the telephone interview (no-choice arm) were as likely to participate as those offered choice of participation mode, whereas in trial 2 those offered a postal questionnaire (no-choice arm) were substantially less likely to participate compared with patients offered choice of participation mode. Considering only patients who indicated to be willing to participate in the SNS, conditional participation rates differed over the two trials as well for the no-choice and choice arms. In trial 1, conditional participation rate was higher in the no-choice arm (for interviews) while it was lower in the no-choice arm for questionnaires in trial 2. Willingness to participate was higher for patients provided with choice of participation mode, although differences with no-choice arms were only significant in trial 1 (no-choice for telephone interviews).

Few previous research seemed to have compared choice of participation mode for telephone interviews and postal questionnaires. Different from Dillman et al. [5] we found that choice of participation mode did enhance participation rate when compared to no choice participation in questionnaires. Some of our results are in accordance with those of Denniston et al. [6] who considered conditional participation rate and found, although not significant, higher conditional participation rates for choice of participation mode compared to no-choice participation in interviews and questionnaires. However, in this study we observed an opposite pattern when comparing choice of mode with the no-choice arm for interviews. In line with Denniston et al. [6], we found that initial willingness to participate was higher for patients provided with choice on participation mode and lowest for patients invited solely for telephone interviews. Reported differences in our study are larger than in previous research comparing choice of participation mode for interviews versus questionnaires [5,6] and questionnaires versus web options [7]. This may have resulted because we recruited patients who were already participating in the TICD-RCT, possibly representing a sample motivated to participate in research.

Results of this study suggest that offering choice of participation mode can enhance participation rates, at least when compared to invitations for participation by a questionnaire. However, this conclusion may seem conflicting with trial 1 in which conditional participation rate was higher in the no-choice arm for interviews. Yet it may be that the participation mode itself created a higher conditional participation rate. Advantages of interviews that may lead to higher participation rates consist, amongst others, of personal contact and opportunity for providing additional explanation and information. Higher participation rates for interviews compared to questionnaires have been described in the literature [18-20]. However, advantages of interviews may have been especially relevant given the topic of the SNS. Although not quantitatively assessed, patients often commented they experienced little burden of their condition or disease and therefore had difficulties relating to questions on persons providing or sharing information on CVRM. An often stated remark was that patients were not in need of information related to CVRM. It may be that advantages of interviews kept these patients in the study while such patients were lost when participating by questionnaires, in which no additional explanation could be provided to patients doubting whether the topic of the research was applicable to their situation. So, it remains possible that conditional participation rates of interviews without choice on participation mode will be different when compared to choice of participation mode for a different research topic.

On the other hand, it may be that practical decisions in the performance of this study reduced participation rates in the choice arms. Patients provided with choice of participation mode but willing to participate by both participation modes, were all sent a questionnaire. Although this approach was chosen for reasons of feasibility, results comparing the no-choice arms of both trials suggest that participation rates could have been higher when patients willing to participate by either mode were interviewed. Interviews may be a less popular mode than questionnaires (willingness to participate in the no choice arm was 73% for interviews in trial 1 vs 86% for questionnaires in trial 2), but they do seem to come with a higher participation rate for those willing to participate in it (participation rates in no choice arm for interviews 66% vs 46% in the no choice arm for questionnaires, and conditional participation rates in no choice arm for interviews 90% vs 53 in no choice arm for questionnaires). So, participation rates in the choice arms of both trials could have been reduced by only using questionnaires as participation mode and could have been higher when interviews were held with patients who were willing to participate by either mode.

Therefore, it may be a valuable strategy to provide choice of participation mode anyhow, using such an approach, optimal participation rates may be attained by 1) providing patients with choice, and 2) usage of participation modes with likely high participation rates, such as interviews, in a maximum number of patients willing to do so.

Limitations of this study consist of the following. First, specific procedures from the research may have influenced outcomes. Due to practical matters, we needed an interval up to a maximum of two months between receipt of accepted invitations and completing interviews and sending questionnaires. It remains unsure which way this may have influenced our results. On one side, participants may have lost their interest or motivation if there is a wider time gap upon deciding to participate and actually doing so. However, participants in the SNS were also participating in the TICD-RCT for which they needed to complete a 20 page (including 87 questions) questionnaire booklet. Therefore, too few time between surveys of both studies may have discouraged patients from participating as well. Another limitation was the provision of only questionnaires in patients willing to participate in the SNS by both participation modes. Third, caution is warranted to generalize findings of this study. As we recruited patients already participating in research, it is possible participants in the SNS represented a more motivated sample to participate in research. The topic of the SNS may limit generalizibility as well. As patients indicated they had difficulties relating to the topic, results may be different for other topics. Finally, concomitant with the applied exclusion criteria of the TICD-RCT, our findings cannot necessarily be generalized to other patient groups, such as those with other chronic diseases or patients with cognitive impairments or poor language skills. Nevertheless, the patient population represents a heterogeneous sample of middle aged and elderly people with one or more chronic diseases.

## Conclusion

Providing choice of participation mode can enhance participation rates, at least when compared to invitations to participate by questionnaires.

### Availability of supporting data

The data set supporting the results of this article is available in the Data Archiving and Networked Services (DANS) repository, http://persistent-identifier.nl/?identifier=urn:nbn:nl:ui:13-gmbq-51.

## Abbreviations

SNS: Social network study
TICD: Tailored implementation for chronic diseases
TICD-RCT: Larger RCT in which the SNS was embedded
CVRM: Cardiovascular risk management
HR: High risk
CVD: Cardiovascular disease
ICPC: International classifications of primary care
RR: Relative risk
GEE: Generalized estimating equation
